# Editorial for the Special Issue on Point-of-Care Devices

**DOI:** 10.3390/mi11040389

**Published:** 2020-04-09

**Authors:** Jane Ru Choi, Kar Wey Yong

**Affiliations:** 1 Centre for Blood Research, Life Sciences Centre, University of British Columbia, 2350 Health Sciences Mall, Vancouver, BC V6T 1Z3, Canada; 2 Department of Mechanical Engineering, University of British Columbia, 2054-6250 Applied Science Lane, Vancouver, BC V6T 1Z4, Canada; 3 Department of Surgery, Faculty of Medicine & Dentistry, University of Alberta, Edmonton, AB T6G 2R3, Canada

Point-of-care (POC) devices, such as paper- and chip-based devices enable the quick collection of patients’ health information to improve healthcare. With the increasing prevalence of chronic and infectious diseases, particularly in the developing countries with poor infrastructure, the use of POC devices is highly desirable. These devices are able to carry out sophisticated tests and give immediate test results in a simple and cost-effective manner. They are particularly useful to detect early infections and monitor underlying diseases which would potentially lead to life-threatening health conditions by enabling immediate administration of appropriate medical treatment. This Special Issue introduced the development of POC devices for various biomedical applications, including cancer screening, viral infection diagnosis and male fertility testing. 

Two comprehensive review articles have discussed the use of microneedle-based devices and electrochemical devices for POC testing. Xie et al. [1] reviewed the selection of materials and fabrication techniques in the development of different types of microneedle-based devices for transdermal sampling and biosensing. The microneedles are made of materials such as silicon, glass, ceramics, metal, polymers and carbohydrate through cutting, etching, photolithography, micromolding and drawing lithography. They are classified into five types: solid, coated, hollow, swellable and dissolving. Different types of microneedle exhibit distinct physicochemical properties, which include biodegradability, swellability, wettability, morphology and mechanical strength. In fact, the coated, hollow and swellable microneedles are commonly used for disease diagnosis and therapeutic monitoring, whereas both solid and dissolving microneedles are widely used for drug delivery application. The microneedle-based devices could be an excellent tool for POC testing in a minimally invasive manner. Mohammadniaei et al. [2] reviewed the development of two-dimensional (2D) material-based electrochemical POC devices for cancer screening. 2D materials such as graphene, transition metal dichalcogenides, Bi_2_Se_3_ and MXene are integrated into chip- and paper-based POC devices for sensitive electrochemical detection of various cancer biomarkers. These electrochemical biosensors hold great potential in cancer diagnosis at the POC due to the requirements of low sample volume, minimum power supply, high sensitivity and low cost. However, some challenges such as weak reproducibility, instability and lack of multiplexing capability are yet to be addressed to translate the technologies into practical applications.

Several research articles have reported the development of chip-, paper-, and nanomaterial-based POC devices for Zika virus detection, sperm quality assessment, and opto-acoustic sensing. Zhu et al. [3] developed “C^3^-system”, a sample-to-answer microfluidic device for POC detection of Zika virus RNA. This device consists of chitosan-modified silicon dioxide capillaries, a pneumatic unit, a heating unit and a smartphone. The positively-charged chitosan allows firm absorption of anionic RNA in acidic condition during RNA extraction while desorbs the RNA in alkaline condition for *in situ* polymerase chain reaction (PCR). A pneumatic unit is used to control the microvalves within the chip for sample and reagent loading, sample lysis, washing and PCR amplification. A smartphone with a fluorescent detection system is used for real-time qualitative detection of Zika virus RNA. The C^3^-system successfully detected Zika virus RNA spiked in saliva at an extremely low concentration (50 transducing units/mL) in 90 minutes. Apart from that, Matsuura et al. [4] developed a wax-printed paper-based colorimetric resazurin assay to evaluate sperm quality. This device successfully detected the reduction of porcine sperm motility for the porcine semen sample treated with 3-Bromopyruvic acid (an enzyme inhibitor that decreases sperm motility) compared to the untreated semen sample. This simple and low-cost paper-based device enables rapid male fertility testing at the POC. In addition, Memisoglu et al. [5] introduced a simple and low-cost procedure of fabricating graphene-based nanomechanical membranes grafted with quantum dots for optoacoustic sensing. Thickness, roughness and deflection distance of the nanomechanical membranes are tuned by the number of graphite exfoliation layer and the ratio of graphene oxide to deionized water to ensure effective emission of vibrating Förster resonance energy transfer (VFRET) signal for target quantification. This nanomaterial-based device could potentially be used for light sensing or photoacoustic-based imaging in the future.

In summary, this special issue has provided unprecedented insights into the use of POC devices for a broad range of biomedical applications. Despite significant advancements, challenges remain in enhancing device performance, stability and reproducibility. While there are many remaining challenges, we believe that there will be more investments in POC research to advance human health. 

## References

[1] Xie L., Zeng H., Sun J., Qian W. (2020). Engineering Microneedles for Therapy and Diagnosis: A Survey. Micromachines.

[2] Mohammadniaei M., Nguyen H.V., Tieu M.V., Lee M.-H. (2019). 2D Materials in Development of Electrochemical Point-of-Care Cancer Screening Devices. Micromachines.

[3] Zhu X., Zhao J., Hu A., Pan J., Deng G., Hua C., Zhu C., Liu Y., Yang K., Zhu L. (2020). A Novel Microfluidic Device Integrated with Chitosan-Modified Capillaries for Rapid ZIKV Detection. Micromachines.

[4] Matsuura K., Wang W.-H., Ching A., Chen Y., Cheng C.-M. (2019). Based Resazurin Assay of Inhibitor-Treated Porcine Sperm. Micromachines.

[5] Memisoglu G., Gulbahar B., Fernandez Bello R. (2020). Preparation and Characterization of Freely-Suspended Graphene Nanomechanical Membrane Devices with Quantum Dots for Point-of-Care Applications. Micromachines.

